# Defective development and microcirculation of intestine in *Npr2* mutant mice

**DOI:** 10.1038/s41598-020-71812-2

**Published:** 2020-09-08

**Authors:** Chizuru Sogawa-Fujiwara, Atsuki Hanagata, Yasuhiro Fujiwara, Yukisato Ishida, Hirotaka Tomiyasu, Tetsuo Kunieda, Hirofumi Nakatomi, Masatoshi Hori

**Affiliations:** 1 Veterinary Pharmacology, Graduate School of Agriculture and Life Sciences, The University of Tokyo, 1-1-1 Yayoi, Bunkyo-ku, Tokyo, Japan; 2 Institute for Quantitative Biosciences, The University of Tokyo, 1-1-1 Yayoi, Bunkyo-ku, Tokyo, Japan; 3 Graduate School of Human Health Sciences, Tokyo Metropolitan University, Minami-Osawa, Hachioji, Tokyo Japan; 4 Veterinary Internal Medicine, Graduate School of Agricultural and Life Sciences, The University of Tokyo, 1-1-1 Yayoi, Bunkyo-ku, Tokyo, Japan; 5 Graduate School of Environmental and Life Sciences, Okayama University, Tsushima-naka, Kita-ku, Okayama, Japan; 6 Department of Neurosurgery, Graduate School of Medicine, The University of Tokyo, 7-3-1 Hongo, Bunkyo-ku, Tokyo, Japan

**Keywords:** Developmental biology, Gastroenterology, Pathogenesis

## Abstract

Intractable gastrointestinal (GI) diseases often develop during infancy. Our group previously reported that natriuretic peptide receptor B (NPR-B)-deficient *Npr2*^*slw/slw*^ mice exhibit severe intestinal dysfunction, such as stenosis and distention, which resembles the dysfunction observed in Hirschsprung’s disease-allied disorders. However, the root cause of intestinal dysfunction and the detailed of pathophysiological condition in the intestine are not yet clear. Here, we report that the intestine of preweaning *Npr2*^*slw/slw *^mice showed bloodless blood vessels, and nodes were found in the lymphatic vessel. Additionally, the lacteals, smooth muscle, blood vessel, and nerves were barely observed in the villi of preweaning *Npr2*^*slw/slw*^ mice. Moreover, intramuscular interstitial cells of Cajal (ICC-IM) were clearly reduced. In contrast, villi and ICC-IM were developed normally in surviving adult *Npr2*^*slw/slw*^ mice. However, adult *Npr2*^*slw/slw*^ mice exhibited partially hypoplastic blood vessels and an atrophied enteric nervous. Furthermore, adult *Npr2*^*slw/slw*^ mice showed markedly reduced white adipose tissue. These findings suggest that the cause of GI dysfunction in preweaning *Npr2*^*slw/slw*^ mice is attributed to defective intestinal development with microcirculation disorder. Thus, it is suggested that NPR-B signaling is involved in intestinal development and control of microcirculation and fat metabolism. This report provides new insights into intractable GI diseases, obesity, and NPR-B signaling.

## Introduction

The intestine has a layered structure and consisting of several tissues including the enteric nervous system (ENS) and interstitial cells of Cajal (ICC). The ENS controls intestinal motility, mucosal secretion, and sensory stimuli^1^. ICC are the pacemakers of the gastrointestinal (GI) tract and act as intermediaries between the ENS and smooth muscle^2,3^. Thus, disturbances of signals from the ENS and/or ICC affect motility and digestion/absorption^4^. Intractable GI diseases are rare, usually develop during infancy, and are often fatal. For example, in patients with Hirschsprung’s disease-allied disorders (HDAD)^5–7^, the ENS is present, yet distension and/or stenosis develop, causing reduced GI motility, a decline in GI transit, and excretory disorders^8,9^. Eventually, these defects lead to bacterial growth, resulting in enteritis and sepsis. However, the cause of GI intractable disease remains unclear.

NPR-B, also known as NPR2 or GC-B, is a transmembrane guanylyl cyclase receptor for the C-type natriuretic peptide (CNP), also known as NPPC. Activation of NPR-B by CNP binding synthesizes intracellular cyclic guanosine-3′,5′-monophosphate (cGMP)^10–13^. CNP also binds to natriuretic peptide receptor C (NPR-C, also known as NPR3 or GC-C)^10,11^, and CNP/NPR-B and/or NPR-C signals most likely act as local autocrine or paracrine factors in several tissues. Previous studies revealed that CNP/NPR-B and/or NPR-C signaling regulates contractility of the gastric antrum and large intestine^14–17^. It was also suggested that the CNP/NPR-B/cGMP signal may be related to ICC activity^18–20^, and vascular remodeling, and blood pressure^21–28^. Furthermore, CNP is also known to be related to adipocyte metabolism^29,30^.

The short-limbed dwarfism (SLW) mouse^31^ has a mutation in *Npr2*, a gene encoding NPR-B, which results in the deletion of the intracellular domain of NPR-B^17^. We previously reported that homozygous (*Npr2*^*slw/slw*^) mice frequently die pre-weaning due to GI dysfunction, such as abnormal retention of gastric milk, a stenosis-like phenotype in the terminal ileum and rectum, and distention of the GI tract by gas^17,32^. In some cases, *Npr2*^*slw/slw*^ mice showed no intestinal abnormality and were fully grown^33^. It has also been recognized that the CNP/NPR-B signal increases cGMP and relaxes the stomach and large intestine^17,32^. These observations indicate the important role of NPR-B in the physiological functions of the GI tract.

The CNP/NPR-B signal evidently regulates vital physiological functions in the GI tract. However, the way that NPR-B deficiency causes GI dysfunction in *Npr2*^*slw/slw*^ mice has not been demonstrated conclusively. Thus, elucidation of the mechanism underlying GI dysfunction in *Npr2*^*slw/slw*^ mice based on the detailed pathophysiological observations will provide valuable insights into GI diseases as well as for understanding CNP/NPR-B signaling.

## Results

### Defective intestinal development in preweaning ***Npr2***^***slw/slw***^ mice

**Figure 1:**
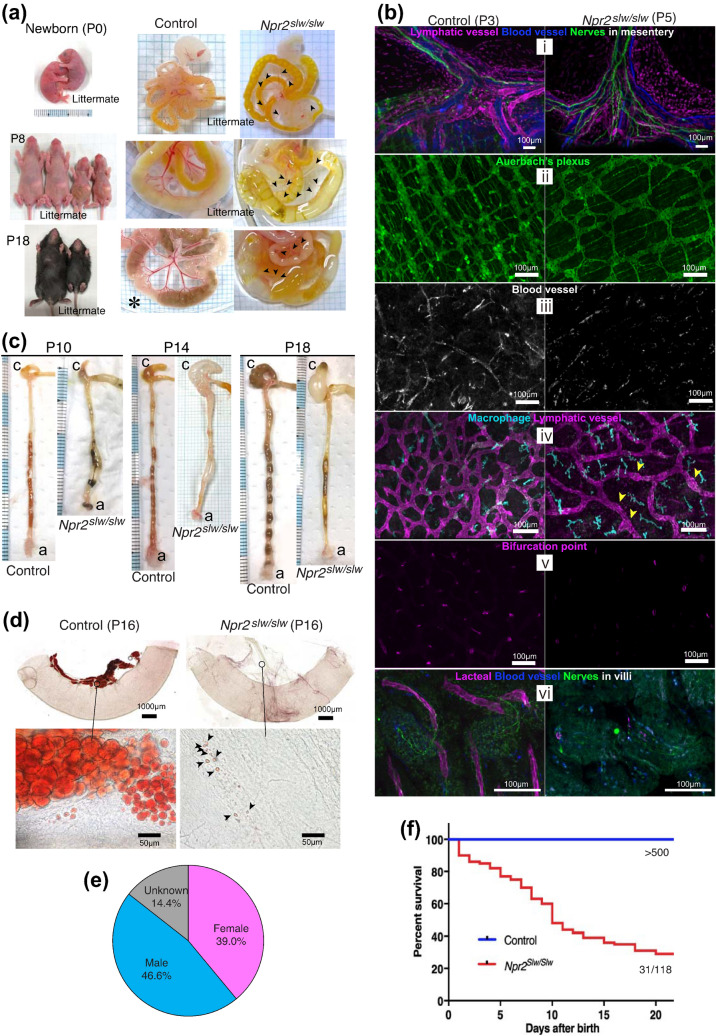
Features of preweaning *Npr2*^*slw/slw*^ mice. (**a**) Appearance and autopsy images of newborn, P8, and P18 mice. The control (left) and *Npr2*^*slw/slw*^ (right) mice. Asterisk indicates non-littermate. Arrowheads indicate mesenteric blood vessel in *Npr2*^*slw/slw*^ mice. (**b**) Whole mount immunostaining of tube specimens (i to v) and flat specimens (vi). i: PECAM (blue), LYVE1 (magenta), and PGP9.5 (green) were to identify blood vessel, lymphatic vessel and LIVE1-positive macrophage, and enteric neurons respectively; ii: PGP9.5 (green) was to identify enteric neurons; iii: PECAM (gray) was to identify blood vessel; iv: LYVE1 (both magenta and cyan) was to identify lymphatic vessel and LIVE1-positive macrophage. Arrowheads indicate small nodes; v: LYVE1 (magenta) was to identify bifurcation point of lacteal; vi: PECAM (blue), LYVE1 (magenta), and PGP9.5 (green) were to identify blood vessel, lacteal, and enteric neurons in villi, respectively. Control (left) and *Npr2*^*slw/slw*^ (right) mice were at P3 and P5, respectively. Bars are 100 µm. (**c**) Autopsy images of large intestine in control (left) and *Npr2*^*slw/slw*^ (right) mice at P10, P14, and P18. (**d**) Whole mount tube specimens of Oil Red-O staining. Control (left) and *Npr2*^*slw/slw*^ (right) mice at P16. Bars are 1 mm or 50 µm. Arrowheads indicate lipids in *Npr2*^*slw/slw*^ mouse. (**e**) Sex ratio of severe GI dysfunction phenotype mice. (**f**) Survival rate of control and *Npr2*^*slw/slw*^ mice showed severe GI dysfunction phenotype until P20.

In most preweaning *Npr2*^*slw/slw*^ mice, abnormal distention was observed in the GI tract (Fig. 1a and Supplementary Fig. 1a). From the time of birth to approximately postnatal day 5 (P5), the small intestine (SI) of control mice, filled with yellow meconium, was covered with multi-branched blood vessels, whereas the amount of blood in blood vessels of *Npr2*^*slw/slw*^ mice was evidently reduced (Fig. 1a, P0 and Supplementary Fig. 1b, P2 arrowheads). At approximately P7 to weaning age, the SI of control mice was filled with milk or fed bolus, and mesenteric blood vessels were more visible and covered with adipose tissue (Fig. 1a, control P8 and P18). However, in *Npr2*^*slw/slw*^ mice, blood in mesenteric blood vessels and adipose tissue was barely observed (Fig. 1a, *Npr2*^*slw//slw*^ P8 and P18 arrowheads). In some cases, SI was filled with viscous bubbles, discolored meconium, or only gas (Supplementary Fig. 1b, P10, P11, and P22). The nerves, blood vessels and lymphatic vessels in the mesentery were present in *Npr2*^*slw/slw*^ mice, as seen in control mice (Fig. 1b i and and Supplementary Fig. 2a). Enteric neurons in the intestinal wall was also present in *Npr2*^*slw/slw*^ mice, as seen in the control (Fig. 1b ii). In *Npr2*^*slw/slw*^ mice, blood vessels in the intestinal wall were disrupted (Fig. 1b iii). Small nodes were observed in lymphatic vessels, and LYVE-1-positive macrophages were slightly larger in *Npr2*^*slw/slw*^ mice than in control mice (Fig. 1b iv). Furthermore, a bifurcated region of lacteals was barely observed (Fig. 1b v). Although villi were formed, their internal contents were defective (Fig. 1b vi), and smooth muscle in villi was barely observed in preweaning age mice (Supplementary Fig. 2b). In contrast, control mice showed branched blood vessels but no lymphatic nodes, and development of these tissues was recognized, albeit at a younger age than *Npr2*^*slw/slw*^ mice (Fig. 1b iii to vi, and Supplementary Fig. 2b). Moreover, a border of stenosis and distention, enteric neurons, was clearly recognized in the intestinal wall of *Npr2*^*slw/slw*^ mice (Supplementary Fig. 2a, magnified image). Meconium and fed bolus were either liquid or absent in the large intestine (LI), and the LI was distended with gas in *Npr2*^*slw/slw*^ mice (Fig. 1c and Supplementary Fig. 1c). In order to visualize adipocytes, we performed Oil Red-O staining of SI, and found intensely stained adipocytes along the mesenteric blood vessels in control mice (Fig. 1d). In contrast, in *Npr2*^*slw/slw*^ mice SI, adipocytes were barely observed (Fig. 1d). Furthermore, in some cases, *Npr2*^*slw/slw*^ mice showed cobweb-like microtubular structures filled with Oil Red-O stained microgranules (Supplementary Fig. 2c and d). Sexual differences were slightly skewed towards males (Fig. 1e); about 74% of *Npr2*^*slw/slw*^ mice developed severe GI dysfunction before P20 (Fig. 1f).

**Figure 2:**
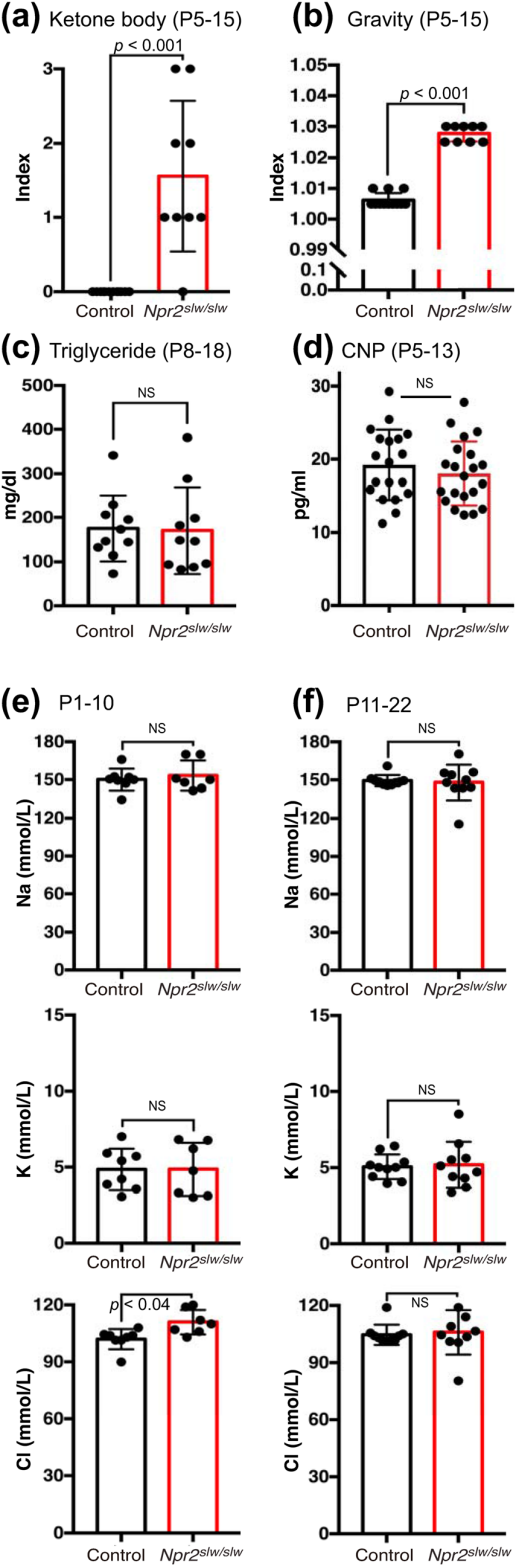
Health condition in preweaning *Npr2*^*slw/slw*^ mice. (**a**) Urinalysis of ketone body and (**b**) gravity. Control (n = 12) and *Npr2*^*slw/slw*^ mice (n = 9). (**c**) Triglyceride level in the blood of control (n = 10) and *Npr2*^*slw/slw*^ mice (n = 10). (**d**) CNP level in the blood of control (n = 19) and *Npr2*^*slw/slw*^ mice (n = 21). (**e**) Electrolyte levels in the blood in P1 to P10 mice. Control (n = 8) and *Npr2*^*slw/slw*^ mice (n = 7). (**f**) Electrolyte levels in the blood in P11 to P22. Control (n = 10) and *Npr2*^*slw/slw*^ mice (n = 10). Bars indicate standard deviation (± SD).

We examined the health condition of *Npr2*^*slw/slw*^ mice that exhibited severe GI disorder. Urinalysis showed a high index for ketone body and gravity (Fig. 2a, b and Supplementary Fig. 3a), and urine of *Npr2*^*slw/slw*^ mice was yellowish (Supplementary Fig. 3b). This indicates that *Npr2*^*slw/slw*^ mice did not absorb enough nutrients and were dehydrated. In some cases, *Npr2*^*slw/slw*^ mice showed a higher protein index than control mice (Supplementary Fig. 3a), indicating renal dysfunction. The levels of triglycerides in the blood were not different between *Npr2*^*slw/slw*^ and control mice (Fig. 2c). We also measured CNP levels in the blood, but no differences were observed between *Npr2*^*slw/slw*^ and control mice (Fig. 2d). Electrolyte levels in the blood were not different between *Npr2*^*slw/slw*^ and control mice at preweaning ages (Fig. 2e, f).

**Figure 3:**
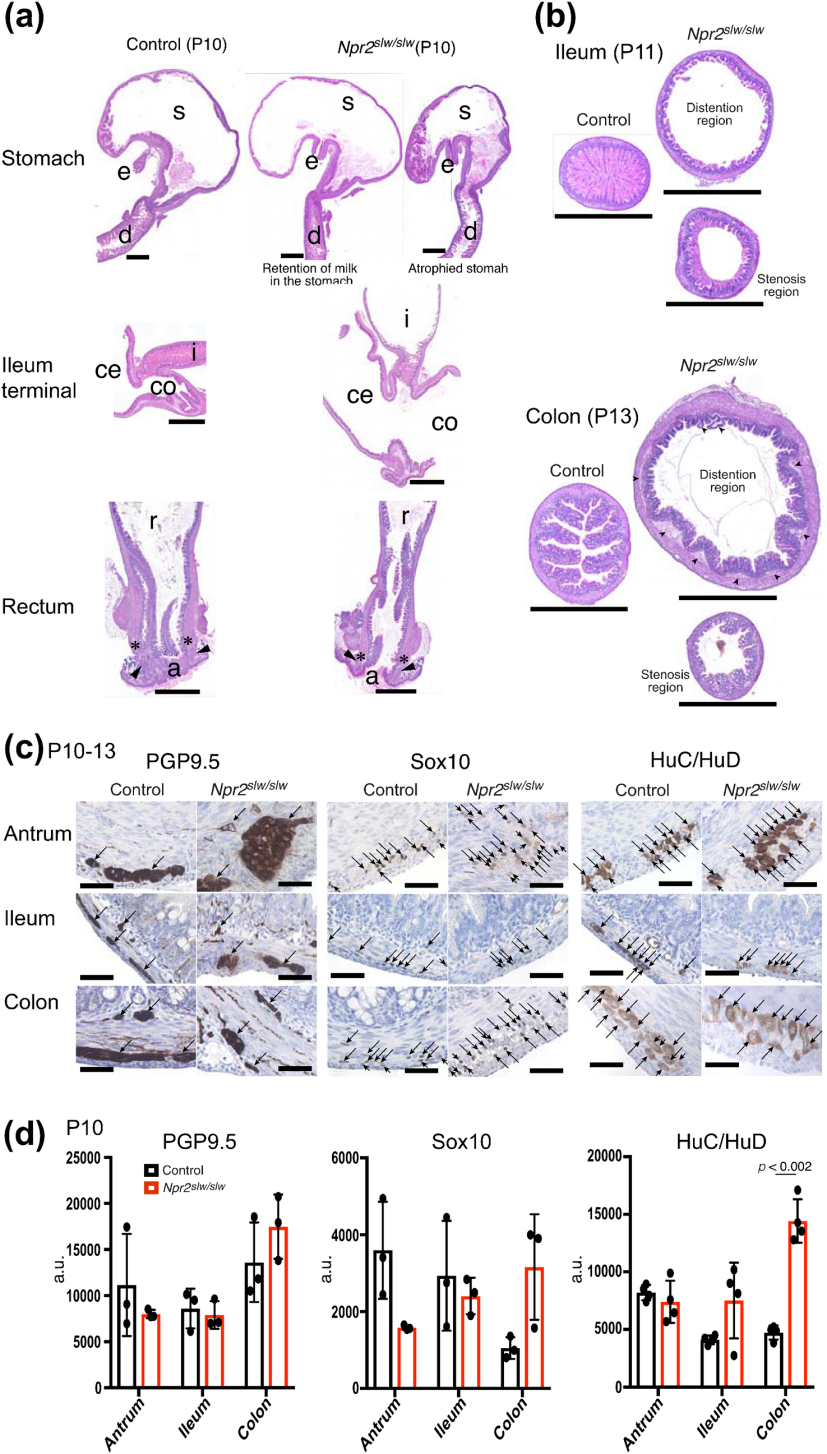
**Histological morphology.** (**a**) HE staining of vertical-sections of stomach to duodenum, terminal ileum area, and rectum to anus (*S* stomach; *e* esophagus; *d* duodenum; *i* ileum; *ce* cecum; *co* colon; *r* rectum; *a* anus) in control and *Npr2*^*slw/slw*^ mice. Asterisks indicate internal anal sphincter and arrowheads indicate external anal sphincter in anus. Bars are 1 mm. (**b**) HE staining of cross-section of ileum and proximal colon in control and *Npr2*^*slw/slw*^ mice. Distention region (upper) and stenosis-like region (lower) in *Npr2*^*slw/slw*^ and corresponding regions in control mice are shown. Bars are 1 mm. (**c**) Localization of pan-neural protein (PGP9.5), enteric glia (Sox10), and ganglion cells (HuC/HuD) in cross-section of antrum, ileum, and proximal colon. Arrows indicate PGP9.5, Sox10, and HuC/HuD-positive cells, respectively. Bars are 50 µm. (**d**) Comparison of protein level of PGP9.5, Sox10, and HuC/HuD by western blotting (n = 3 each for control and *Npr2*^*slw/slw*^ mice). Columns indicate mean and bars indicate ± SD. a.u. is an arbitrary unit.

We assessed the morphology of regions of stenosis-like conditions such as the antrum, ileum terminal, and rectum. We compared the histological morphology of these regions between control and *Npr2*^*slw/slw*^ mice at P10. From stomach to duodenum, gastric distention and atrophy were seen in some *Npr2*^*slw/slw*^ mice (Fig. 3a stomach and Supplementary Fig. 4a stomach). In the regions before and behind the terminal ileum, the lumen of the terminus of control mice remained unconnected. In contrast, the distal part of ileum, cecum, and proximal colon of *Npr2*^*slw/slw*^ mice were distended by gas, whereas the terminal ileum remained closed (Fig. 3a ileum terminal and Supplementary Fig. 4a ileum terminal). From the rectum to the anus, there were no morphological differences between control and *Npr2*^*slw/slw*^ mice (Fig. 3a rectum and Supplementary Fig. 4a rectum). Additionally, *Npr2*^*slw/slw*^ mice showed immature villi and mucosal epithelium in SI and LI (Fig. 3b), and intestinal edema in the distention region of the LI (Fig. 3b arrows head). We further examined the localization and morphology of enteric neurons by immunohistochemical staining of the antrum, ileum, and proximal colon using antibodies against PGP9.5, a pan-neuronal marker, Sox10, an enteric glial marker, and HuC/HuD, a ganglion cell marker. No differences were found in neuronal and enteric glial cells. However, ganglion cells appear to be slightly larger in *Npr2*^*slw/slw*^ mice (Fig. 3c and Supplementary Fig. 4c). Additionally, western blot analysis confirmed that expression levels of PGP9.5, Sox10, and HuC/HuD were not significantly different between control and *Npr2*^*slw/slw*^ mice, except for HuC/HuD in the colon, which were higher in *Npr2*^*slw/slw*^ mice (Fig. 3d).

**Figure 4:**
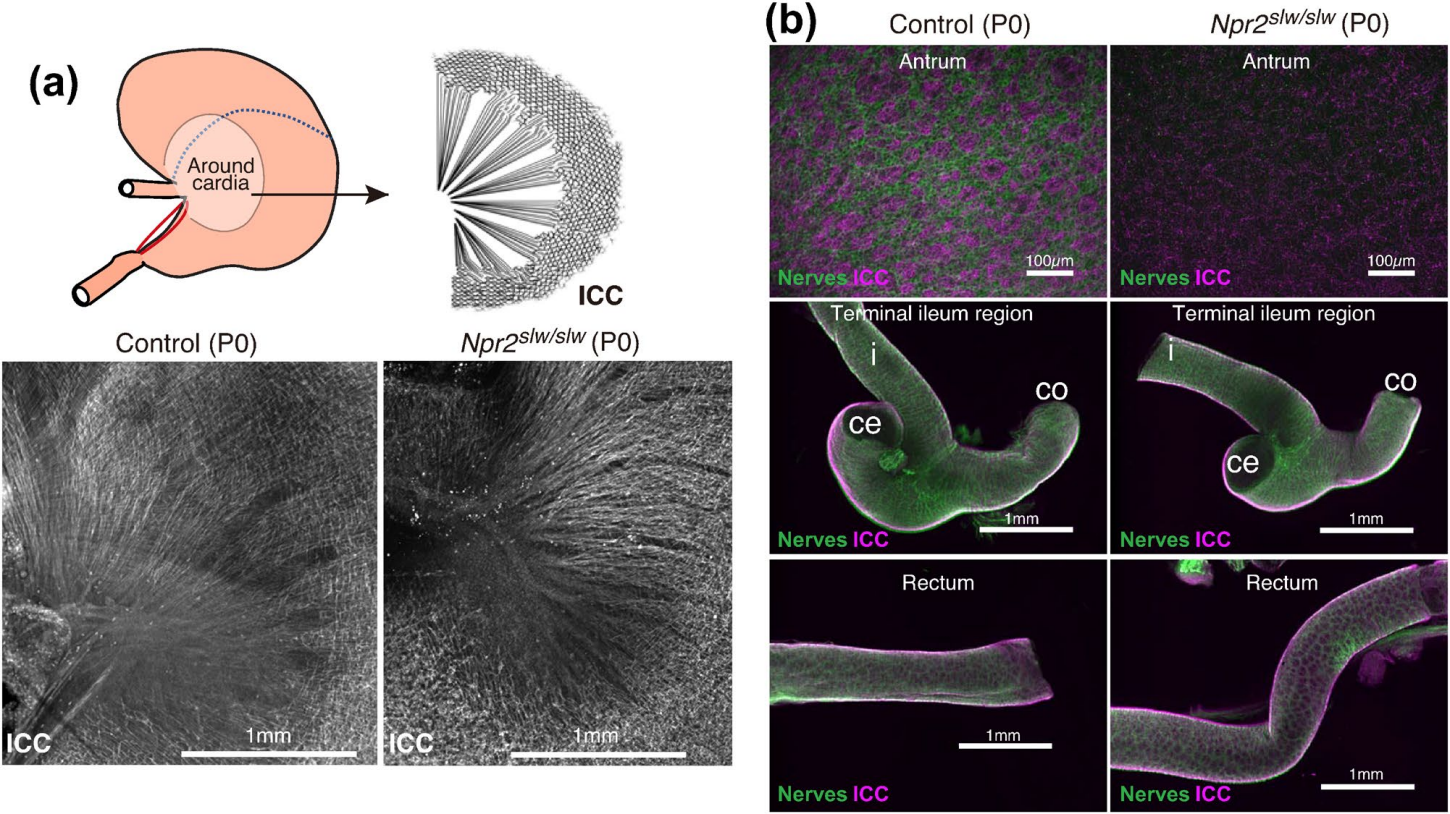
Enteric neurons and ICC in P0 mice. (**a**) Whole mount immunostaining of tube specimens. Morphological characteristics of ICC in around cardia. CD117 (gray) was to identify ICC. Bars are 1 mm. (**b**) Whole mount immunostaining of tube specimens for PGP9.5 (green) and CD117 (magenta) were to identify enteric neurons and ICC, respectively. Control (left) and *Npr2*^*slw/slw*^ (right) mice. *i* ileum; *ce* cecum; *co* colon. Bars are 100 µm and 1 mm as indicated.

### Morphology of ICC and enteric neurons in preweaning ***Npr2***^***slw/slw***^ mice

**Figure 5:**
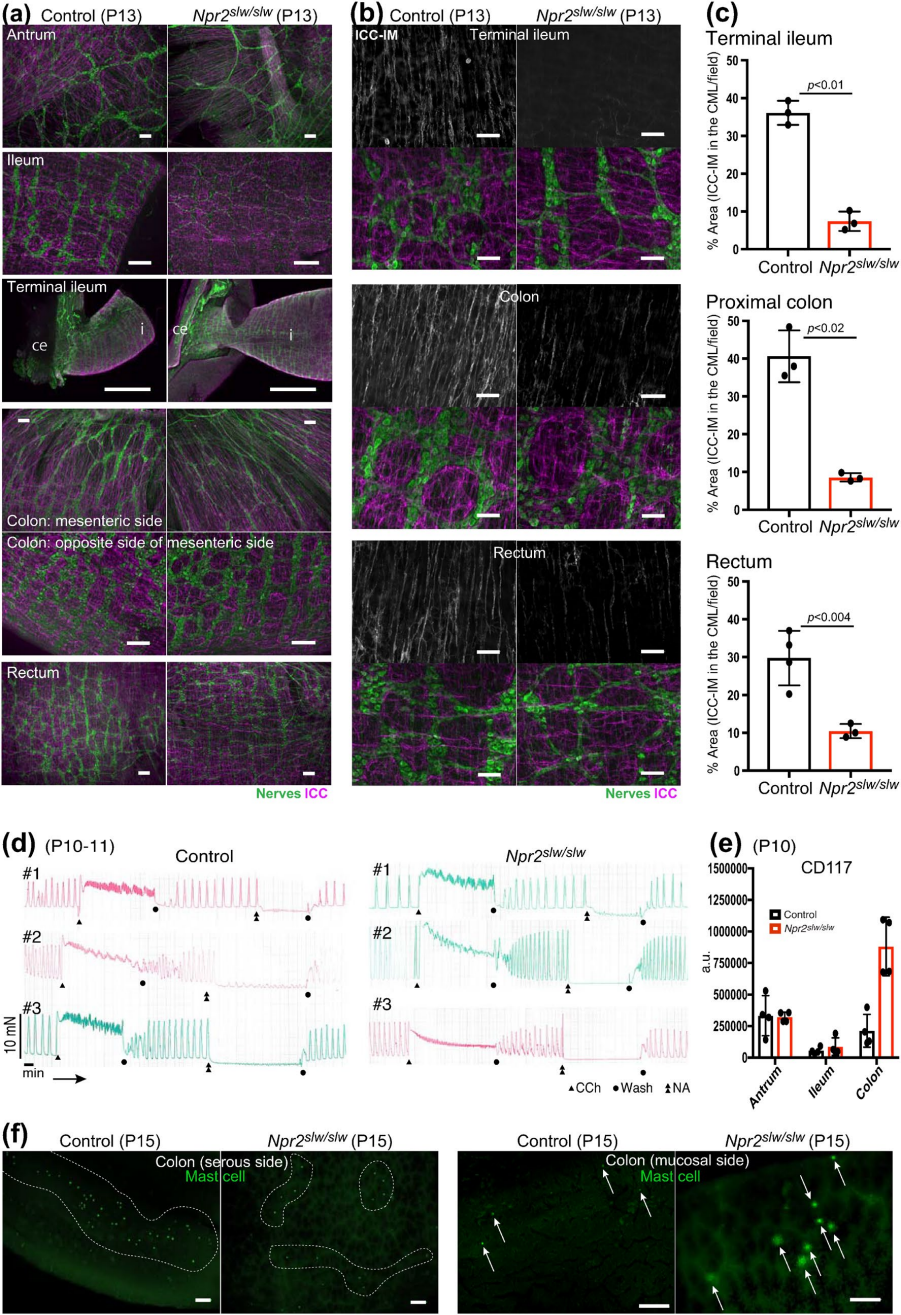
Enteric neurons and ICC in 2 weeks mice and motility. (**a**) Whole mount immunostaining of tube specimens for PGP9.5 (green) and CD117 (magenta) were to identify enteric neurons and ICC, respectively in control (left) and *Npr2*^*slw/slw*^ (right) mice. i: ileum; ce: cecum. Bars are 100 µm (images with short bars were captured using a 10 × objective lens; images with long bars were captured using a 20 × objective lens). Bars in terminal ileum are 1 mm. (**b**) Whole mount immunostaining of flat specimens for PGP9.5 (green) and CD117 (gray or magenta) were to identify enteric neurons and ICC in terminal ileum, proximal colon, and rectum. Upper row, Gray signal indicates ICC-IM in the circular muscle layer. Lower rows show the same position of upper row. enteric neurons (green) and ICC (magenta) were stacked from circular muscle layer to longitudinal muscle layer. Bars are 50 µm. (**c**) Columns indicate the area of ICC-IM in image field of terminal ileum, proximal colon, and rectum (n = 3 or 4 each for control and *Npr2*^*slw/slw*^ mice, bars indicate ± SD). (**d**) Spontaneous rectal motility and response of smooth muscle by muscarinic and adrenergic agonists carbachol and noradrenaline. Triangles indicate the time of addition of 5 µM carbachol. Double triangles indicate the time of addition of 3 µM noradrenaline. Circles indicate the time of flushing with physiological salt solution (n = 3 each for control and *Npr2*^*slw/slw*^ mice). *CCh* carbachol; *NA* noradrenaline. (**e**) Comparison of protein level of CD117 by western blotting (n = 3 each for control and *Npr2*^*slw/slw*^ mice). Columns indicate mean and bars indicate ± SD. a.u.: arbitrary unit. (**f**) Whole-mount labelling of colon flat specimens using FITC-avidin (green) was to identify mast cells. Serous side (left panels) and mucosal side (right panels) in control (n = 2) and *Npr2*^*slw/slw*^ mice (n = 2). Mast cells are located within enclosed dashed circles or indicated by arrows. Bars are 100 µm.

We examined the newborn status of ICC and enteric neurons, because in *Npr2*^*slw/slw*^ mice, milk was seen in the stomach. To examine whether enteric neurons and ICC are affected in *Npr2*^*slw/slw*^ mice, we comprehensively analyzed morphology of enteric neurons and ICC. In *Npr2*^*slw/slw*^ mice at P0, around cardia, CD117-positive ICC was organized in a fan-like direction in both control and *Npr2*^*slw/slw*^ mice (Fig. 4a). Although enteric neural crest-derived cells are known to migrate from the upper to lower regions of the GI tract^1^, PGP9.5-positive enteric neurons and ICC were immature in antrum as compared with that of control mice, while they were normally present in the ileum to the rectum in both control and *Npr2*^*slw/slw*^ mice (Fig. 4b and Supplementary Fig. 5a and b). Subsequently, at around 2 weeks of age, many *Npr2*^*slw/slw*^ mice developed severe GI phenotype at this time. Enteric neurons and ICC were present in all regions in both mice (Fig. 5a) but enteric neurons were severely disrupted in the abnormally distended region in *Npr2*^*slw/slw*^ mice (Fig. 5a, ileum). However, development and elongation of enteric neurons and ICC in the antrum and ileum terminal to rectum showed no differences between control and *Npr2*^*slw/slw*^ mice (Fig. 5a). We measured the percent immunostained area for intramuscular ICC in the circular muscle layers (ICC-IM), terminal ileum, proximal colon, and rectum, and it was revealed that there were significantly fewer ICC-IM in *Npr2*^*slw/slw*^ mice (Fig. 5b,c). In many cases, enteric neurons were present, not only ICC-IM but also ICC-MY, an Auerbach’s plexus layers, and ICC-IM in the longitudinal muscle layers of *Npr2*^*slw/slw*^ mice were underdeveloped in LI compared with those of control mice (Supplementary Fig. 6a and b). We examined the reactivity of the intestinal smooth muscle using muscarinic and adrenergic agonists carbachol and noradrenaline. The result indicated that contractile and relaxant responses of smooth muscle to carbachol and noradrenaline were not different between control and *Npr2*^*slw/slw*^ mice (Fig. 5d). Unexpectedly, western blot analysis of the whole layered GI tract showed that the expression level of CD117 in the LI was significantly higher in *Npr2*^*slw/slw*^ mice than that in control mice, whereas no difference was shown in the stomach and ileum between control and *Npr2*^*slw/slw*^ mice (Fig. 5e). Because CD117 is also a marker of mast cells, we examined mast cells of colon using FITC-avidin that is known to label mast cells^34^. Whole-mount labeling revealed mesenteric mast cells were fewer in *Npr2*^*slw/slw*^ mice than those in control. However, mucosal mast cells of *Npr2*^*slw/slw*^ mice were often observed and apparently larger whereas those in control mice were rarely observed (Fig. 5f).

**Figure 6:**
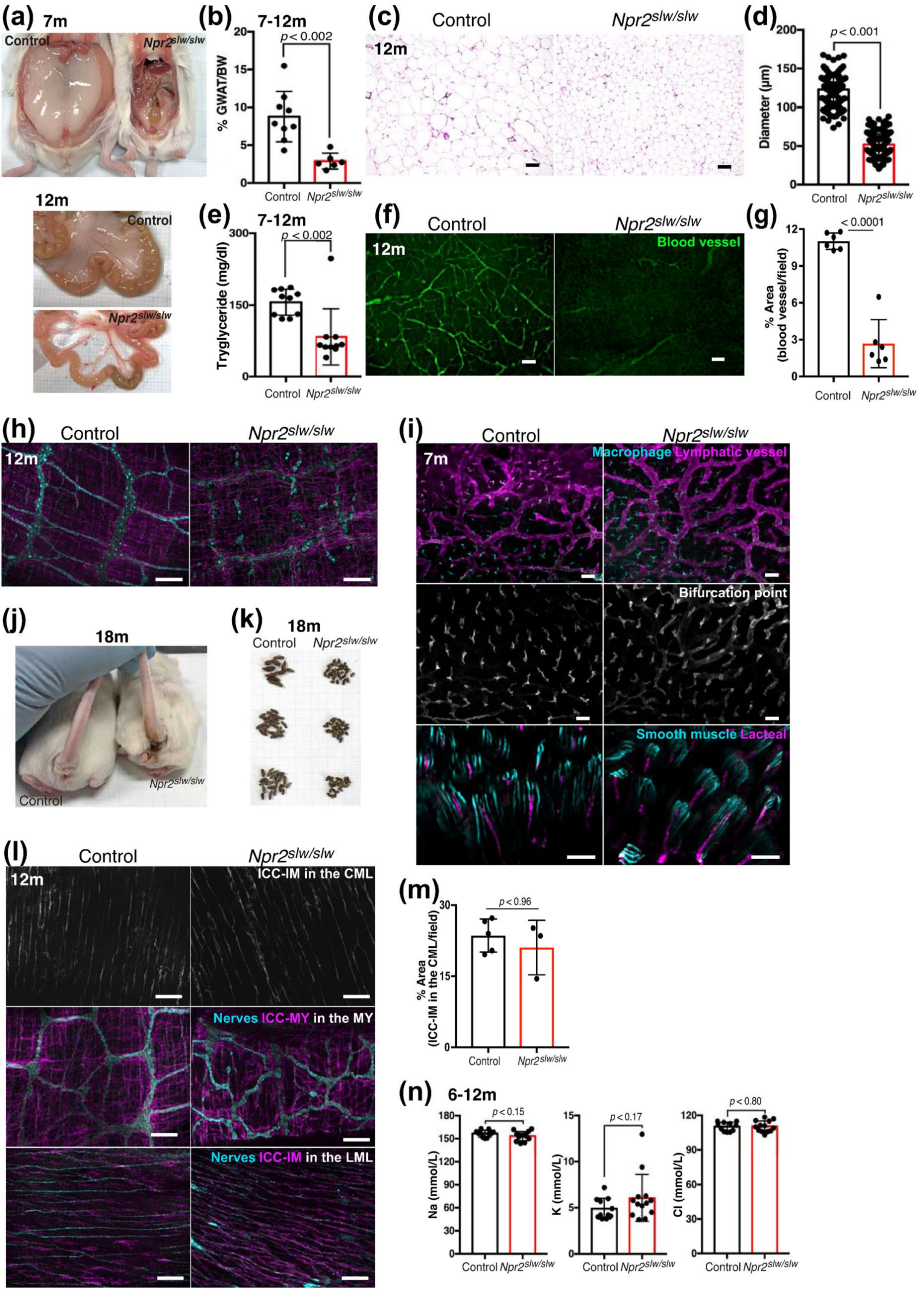
Pathological analysis of adult *Npr2*^*slw/slw*^ mice. All immunostaining images are from whole mount flat specimen. m represents month. (**a**) Autopsy images of GWAT in control (left) and *Npr2*^*slw/slw*^ (right) mice and mesenteric white adipose tissue in control (upper row) and *Npr2*^*slw/slw*^ (lower row) mice. (**b**) Ratio of GWAT per body weight (BW) in control (n = 9) and *Npr2*^*slw/slw*^ mice (n = 6). (**c**) HE staining of GWAT in control (left) and *Npr2*^*slw/slw*^ (right). Bars are 100 µm. (**d**) Diameter of adipocyte in GWAT in control and *Npr2*^*slw/slw*^ mice. (**e**) Triglyceride level in the blood of control (n = 10) and *Npr2*^*slw/slw*^ mice (n = 10). (**f**) PECAM (green) was to identify blood vessel in ileum at opposite side of mesenteric side. Control (left) and *Npr2*^*slw/slw*^ (right) mice. Bars are 100 µm. (**g**) Columns indicate the percent area of PECAM positive signal in image field (n = 6 each for control and *Npr2*^*slw/slw*^ mice images, shown as mean ± SD). (**h**) PGP9.5 (cyan) and CD117 (magenta) were to identify enteric neurons and ICC in ileum of control (left) and *Npr2*^*slw/slw*^ (right) mice. Bars are 100 µm. (**i**) LYVE1 (magenta, cyan, and gray) was to identify lymphatic vessel, macrophage, lacteal in ileum. aSMA (cyan) was to identify smooth muscle in villi. Upper row: lymphatic vessel and macrophage, respectively; Middle row: bifurcation point of lacteal; Lower row: lacteal and smooth muscle of villi. Bars are 100 µm. (**j**) Dyschezia in control and *Npr2*^*slw/slw*^ mice. (**k**) Stools of control and *Npr2*^*slw/slw*^ mice were obtained from non-littermate. (**l**) PGP9.5 (cyan) and CD117 (gray or magenta) were to identify enteric neurons and ICC in rectum. Upper row: Gray signal indicates ICC-IM in the CML; Middle rows show the ICC merged with enteric neurons in the MY; Lower rows show the ICC merged with enteric neurons in the LML. Bars are 100 µm. CML, circular muscle layer; MY, layer of Auerbach's plexus; LML, longitudinal muscle layer. (**m**) Columns indicate the percent area of ICC-IM in the CML per image field in rectum (n = 5 and 3 each for control and *Npr2*^*slw/slw*^ mice, shown as mean ± SD). (**n**) Electrolyte levels of serum in adult mice. Control: n = 11; *Npr2*^*slw/slw*^ mice: n = 13.

### Adult ***Npr2***^***slw/slw***^ mice

Approximately 26% of *Npr2*^*slw/slw*^ mice survived to adulthood, defined as mice over 6 months of age. Interestingly, gonadal white adipose tissue (GWAT) and mesenteric white adipose tissue of adult mice were evidently lower in *Npr2*^*slw/slw*^ mice than those in the control mice (Fig. 6a and Supplementary Fig. 7a and b). The ratio of GWAT per body weight was significantly smaller in *Npr2*^*slw/slw*^ mice than that in the control mice (Fig. 6b). Adipocytes were also smaller in *Npr2*^*slw/slw*^ mice than those in the control mice (Fig. 6c), and the diameter of these adipocytes was significantly reduced in *Npr2*^*slw/slw*^ mice compared with that in the control mice (Fig. 6d). The level of triglycerides in the blood was significantly lower in *Npr2*^*slw/slw*^ mice than that in the control mice (Fig. 6e). Additionally, we frequently observed hypoplastic blood vessels with swollen areas in the SI of *Npr2*^*slw/slw*^ mice (Supplementary Fig. 7c). Significantly fewer branches of intestinal blood vessels were observed in *Npr2*^*slw/slw*^ mice than in the control mice (Fig. 6f, g, and Supplementary Fig. 7d). Further, both normal and atrophied enteric neurons and ICC were observed in the SI of *Npr2*^*slw/slw*^ mice (Fig. 6h and Supplementary Fig. 7e). The lymphatic vessel, bifurcation point of the lacteal, and lacteals with smooth muscle in villi were developed in both control and *Npr2*^*slw/slw*^ mice (Fig. 6i). Additionally, they occasionally suffered obstructed defecation and debilitation (Fig. 6j and Supplementary Fig. 7f.), and stools of aged *Npr2*^*slw/slw*^ mice were smaller than those of the control mice (Fig. 6k). We thus analyzed the enteric neurons and ICC morphology of LI, and found that ICC including ICC-IM was developed in *Npr2*^*slw/slw*^ and control mice (Fig. 6l for CML and LML, Fig. 6m for ICC-IM in the CML), while enteric neurons of *Npr2*^*slw/slw*^ mice were atrophied in many cases (Fig. 6l, MY), but some regions showed normal enteric neurons (Supplementary Fig. 7g). Electrolyte levels in serum were not different between control and *Npr2*^*slw/slw*^ mice (Fig. 6n).

## Discussion

In our previous studies, we focused on smooth muscle function as a cause of GI dysfunction in *Npr2*^*slw/slw*^ mice^17,32^. However, the present study suggested that distention of *Npr2*^*slw/slw*^ mice was not only attributed to stenosis but also to distension caused by defective development and circulation. It is likely that NPR-B-deficient *Npr2*^*slw/slw*^ mice lost the ability to excrete waste, exchange gas, and absorb nutrients, which in turn caused viscous bubbles and corrosion of GI contents. However, although lacteals were not fully developed, the level of triglycerides in the blood was not significantly different compared with that in control mice at preweaning age. This may indicate that lipid absorption is not mediated by lacteal in lactation-period mice. Unlike lymphangiogenesis defective model mice (including *Prox1*+/− mouse^35^ and *vegfc* deleted-mouse^36^), pups that showed intra-abdominal leakage of chylus, *Npr2*^*slw/slw*^ mice did not exhibit such leakage. This is probably indicative of an intact lymphatic vessel itself.

CNP/NPR-B is known to regulate the vascular system. Given that the intestine of *Npr2*^*slw/slw*^ pups showed bloodless blood vessels, and that adults showed hypoplastic intestinal blood vessels, it strongly suggests that NPR-B plays an important role in the GI tract’s vascular system, including the bloodstream. Recent studies have suggested the important role of NPR-B and its ability to produce cGMP in the pericytes of microvessels reducing blood pressure^27,28^. Additionally, it was recently reported that CNP/NPR-C, rather than the CNP/NPR-B signal, plays an important role in angiogenesis and vascular remodeling^23–26^. This implies that NPR-B regulates blood pressure and blood flow, and in contrast, NPR-C regulates endothelial cells in the vascular system, which is involved in vascular remodeling.

Our comparative analysis between preweaning and adult mice clearly showed developmental delay of enteric neurons and/or ICC in the antrum and large intestine in *Npr2*^*slw/slw*^ mice. This is probably the cause of abnormal retention in gastric milk and distention of the LI in pups. Additionally, high level of CD117 by western blot in the LI in *Npr2*^*slw/slw*^ mice may be indicative of higher number of ICC-MY or enhanced proliferation of mucosal mast cells. However, *Npr2*^*slw/slw*^ mice retained normal spontaneous motility and normal smooth muscle response by carbachol and noradrenaline ex vivo. Similarly, another lineage of NPR-B-mutant *Npr2*^*cn/cn*^ showed defective axonal bifurcation in spiral ganglion neurons and disorganized cochlear nuclei, and yet showed a normal sensitivity and response to sounds^37^.

This study also provides new findings regarding the maintenance of enteric neurons. Adult mice appeared healthy in many ways but tended to become debilitated earlier than controls. Indeed, atrophy of enteric neurons was observed in aged *Npr2*^*slw/slw*^ mice. The hypoplastic blood vessels are probably related to neural maintenance, and CNP/NPR-B may play an important role in the neurovascular unit in the GI tract. Furthermore, adult male *Npr2*^*slw/slw*^ mice develop erectile dysfunction^33^, probably also related to neurovascular function.

Overexpression of CNP specifically in endothelial cells or adipocytes (CNP-Tg mice) decreased mesenteric, epididymal, and subcutaneous fat, and prevented hypertrophy of adipocytes with a high-fat diet, whereas mice fed a standard diet showed no difference in adipocyte weight^29,30^. Intriguingly, adult NPR-B-deficient *Npr2*^*slw/slw*^ mice, in which lacteal developed normally, exhibited reduced perigonadal and mesenteric fat, both. This phenotype resembles that of CNP-Tg mice in which adipose tissues or adipocytes are reduced. This may imply an effect of the CNP/NPR- “C” function. Alternatively, the phenotype of *Npr2*^*slw/slw*^ may indicate that NPR-B is involved in the synthesis and/or storage of triglycerides, or that NPR-B negatively regulates energy metabolism. Additionally, although GI dysfunction phenotypes have not been reported in other CNP/NPR-B mouse strains, it remains unclear why only *Npr2*^*slw/slw*^ mice develop a unique GI phenotype that includes abnormalities of blood and lymphatic vessels.

Recent studies have suggested that CNP is related to inflammation. For example, CNP plasma concentrations increased in humans and rats with organ dysfunction caused by sepsis-related inflammation^38,39^. However, CNP plasma levels were indistinguishable between control and *Npr2*^*slw/slw*^ mice. The intestines of *Npr2*^*slw/slw*^ mice did not turn red, and there were no signs of inflammation in *Npr2*^*slw/slw*^ mice. Additionally, the level of electrolytes was indistinguishable between control and *Npr2*^*slw/slw*^ mice. Indeed, the loss of NPR-B possibly has no effect on electrolyte concentrations in circulation.

In conclusion, our findings will help to understand the role of NPR-B in the GI tract and its microcirculatory system. Moreover, the observations of this study could be a clue to the cause of obesity and cryptogenic GI intractable disease.

## Methods

### Animal

An inbred SLW strain (*Npr2*^*slw*^: white coat) with a mixed genetic background of ddY (white coat) and C57BL/6 J (black coat) was obtained from the National Institute of Radiological Sciences (Chiba, Japan). To generate a congenic line C57BL/6J-*Npr2*^*slw*^ (B6-*Npr2*^*slw*^), the inbred strain was backcrossed to the C57BL/6JJsslc (Japan SLC, Inc., Shizuoka, Japan) over seven generations, and subsequently heterozygous littermates were mated. An F1 SLW line was created from a mating between heterozygous inbred *Npr2*^*sl*w^ and B6-*Npr2*^*slw*^ mice. An F2 SLW line was created from a mating between F1 heterozygotes of inbred and C57BL/6JJsslc mice. In this study, we used inbred, F1, and F2 lines, all of which showed the same phenotype. All mice were maintained under a standard 12-h light/dark condition. Either heterozygotes or wild-type mice were used for comparison (referred to as controls); homozygotes are referred to as *Npr2*^*slw/slw*^. Followed by injecting an excess amount of a combined anesthetic containing 7.5% Domitor, 8% Midazolam and 10% Butorphanol tartrate in saline subcutaneously, all specimens were prepared.

All animal experiments were carried out in accordance with institutional guidelines regarding animal care and handling, and the experimental protocol was approved by the Institutional Animal Care and Use Committee of the University of Tokyo (Approval number P17-27H02).

### Intestinal pharmacological response study

A pharmacological response study was performed as described previously^17^, with some modifications. Briefly, the rectum of control and *Npr2*^*slw/slw*^ mice was collected and placed in physiological salt solution (PSS). Rectal strips with a whole layer were suspended longitudinally in 20 mL of PSS at 37 °C in an atmosphere containing 95% O_2_ and 5% CO_2_. After equilibration, each strip was exposed to 65.4 mmol potassium solution to obtain a stable contractile response. Subsequently, carbachol and noradrenaline were added to PSS to reach final concentrations of 5 µM and 3 µM, respectively. Contractile and relaxant movement profiles were recorded.

### Quantification of protein expression level

Expression levels of marker proteins in the mouse GI tissue samples were measured by a Simple Western assay using a Jess (ProteinSimple, Santa Clara, CA, USA). Stomach (corpus to antrum), ileum (distal region), and colon (proximal region) were homogenized with RIPA buffer (182–02,451, Fujifilm Wako Pure Chemical Corporation, Osaka, Japan) containing a protease inhibitor cocktail (P8340, Sigma Aldrich, MO, US), and loading samples were prepared using a 10 × sample buffer (042–195, ProteinSimple) containing EZ Standard pack 1 (PS-ST01EZ-8). A protein normalization module (DM-TP02, ProteinSimple) was used for protein normalization according to the manufacture’s procedure. Samples were run on a Jess Separation Capillary Cartridge (SM-W004, ProteinSimple). Primary antibodies used were rabbit polyclonal anti-PGP9.5 antibody (ADI-905–520-1, ENZO Life Sciences, Farmingdale, NY, US) for enteric nervous, mouse monoclonal anti-HuC/HuD antibody (A21271, Thermo Fisher, Waltham, MA, USA) for the ganglion cells, mouse monoclonal anti-Sox10 antibody (sc0365692, Santa Cruz Biotechnology, Santa Cruz, CA, USA) for intestinal glial cells, and goat polyclonal anti-CD117/c-kit antibody (AF1356, R&D systems) for ICC. Antibodies were diluted in an antibody diluent buffer (042–203, ProteinSimple) that was also used as a blocking buffer. Secondary antibodies used were anti-rabbit IgG antibody (042–206, ProteinSimple) for PGP9.5, anti-mouse IgG antibody (042–205, ProteinSimple) for HuC/HuD and Sox10, and anti-goat IgG antibody (705–035-147, Jackson ImmunoResearch Inc., West Grove, PA, USA) for CD117/c-kit. Chemiluminescent signals were detected using luminol-S (043–311, ProteinSimple) and peroxide (043–379, ProteinSimple). Data were analyzed using a software Compass for SW 4.0 (ProteinSimple).

### Histology and immunostaining

Whole GI tissue was removed mesentery and fixed in Bouin’s solution (85% saturated picric acid, 3.7% formaldehyde [10% 37% formaldehyde], and 5% glacial acetic acid) at room temperature (RT) for 2 h, and then immersed in 50% ethanol for 2 days. The specimens were trimmed, dehydrated and embedded in paraffin. Six-µm-thick sections were cut, placed on glass slides, and subjected to hematoxylin and eosin (HE) staining.

For immunostaining with diaminobenzene (DAB), antigen retrieval was performed by incubating in 10 mmol/L sodium citrate (pH 6.0) that contained 0.05% Tween-20 for 15 min at 95 °C, and gradually cooling to RT. The sections were washed in distilled water, immersed in TBS (50 mmol Tris–HCl, 150 mmol NaCl, and 2 mmol KCl), and then incubated in peroxidase-blocking solution (3% H_2_O_2_ in methanol) for 10 min at RT, followed by washing three times in TBS for 5 min at RT. The sections were then blocked with a non-specific blocking reagent (X0909, Dako, Glostrup, Denmark) for 10 min at RT, and incubated 2 h with the primary antibody at RT. The antibodies used were rabbit polyclonal anti-PGP9.5 antibody (ENZO Life Sciences, 1:200 dilution) for enteric nervous, mouse monoclonal anti-HuC/HuD antibody (Life Technologies Corporation, 1:100 dilution) for the ganglion cells, and mouse monoclonal anti-Sox10 antibody (Santa Cruz Biotechnology, 1:20 dilution) for intestinal glial cells. After incubation, the sections were washed with TBS three times for 5 min at RT and incubated with the secondary antibody for 1 h at RT. The secondary antibodies used were: ENVISION + rabbit HRP (K4002, Dako) for PGP9.5 and ENVISION + mouse HRP (K4000, Dako) for HuC/HuD and Sox10. After washing three times with TBS for 5 min at RT, the sections were stained with DAB (K3468, Dako) for 1–2 min at RT and then washed with distilled water. The nuclei were stained with hematoxylin.

### Whole mount immunofluorescence and fluorescence staining

For tube specimen, whole GI tissue with mesentery was fixed in 10% buffer formalin for overnight, and then immersed in TBS for overnight. The specimens were trimmed and washed with TBS. Subsequently, specimens were incubated in peroxidase-blocking solution for bleaching and demembranation for 30 min at RT. Followed by washing with TBS three times for 30 min at RT. The specimens were incubated with non-specific blocking reagent for 30 min at RT, and then reacted with the primary antibodies for 1–2 days at 4 °C on a shaker. The primary antibodies used were: rabbit polyclonal anti-PGP9.5 antibody (ENZO Life Sciences, 1:200 dilution) for enteric nervous, goat polyclonal anti-CD117/c-kit antibody (AF1356, R&D systems, 1:100 dilution) for ICC, rabbit polyclonal anti-alpha smooth muscle actin (aSMA) antibody (ab5694, Abcam, Cambridge, UK, 1:100 dilution) for smooth muscle, goat polyclonal LYVE-1 antibody (AF2125, R&D systems, 1:100 dilution) for lymphatic vessel, mouse monoclonal anti-PECAM antibody (sc-376764, Santa Cruz, CA, US, 1:20 dilution) for vascular endothelial cells, and FITC-avidin (434411, Thermo Fisher, 1:1,000 dilution) for mast cells. Each specimen was washed with TBS three times for 30 min at RT, and then incubated with the secondary antibodies overnight at 4 °C on a shaker in a dark condition. The secondary antibodies used were: donkey rabbit IgG H&L Alexa flour 488 (A21206, Thermo Fisher, 1:500 dilution) for PGP9.5 and aSMA, donkey goat IgG H&L 594 (A11058, Thermo Fisher, 1:500 dilution) for CD117/c-kit and LYVE-1, goat mouse IgG H&L Alexa flour 488 (A11001, Thermo Fisher, 1:500 dilution) for PECAM, and donkey goat IgG H&L Alexa flour 350 (A21081, Thermo Fisher, 1:500 dilution) for PECAM. Each specimen was washed with TBS three times for 30–60 min at RT, replaced with 50% glycerol in TBS on a glass bottom dish and covered with a cover glass.

For flat specimen, trimmed tube specimen as prepared above was cut longitudinally, immunostained, mounted with 50% glycerol TBS on a glass slide or glass bottom dish, and covered with a cover glass.

### Oil Red-O staining

The whole GI tract with mesentery was fixed in 10% formalin overnight at 4 °C, and washed in tap water at RT for 1 h; then the specimens were trimmed and immersed in TBS until staining at 4 °C. The specimens were washed in doubly distilled water (DDW) for 3 min, immersed in 60% isopropyl alcohol for 2 min, and then immersed in filtered 60% Oil Red-O (4,049–1, Muto Pure Chemicals Co., Ltd, Tokyo, Japan) at 37 °C for 15 min. The specimens were washed in 60% isopropyl alcohol for 2 min and in DDW for 2 min.

### Microscopy and acquisition

Stained images were acquired using BZ-X710 (Keyence, Osaka, Japan). HE and DAB stained sections were stacked and tiled. Wholemount fluorescent-immunostained and Oil Red-O stained whole-mount specimens were acquired using a BZ-H3XF/Sectioning Module. Unstained intestinal specimens were photographed using a stereo microscope (SZX16, Olympus).

### Measurement of serum triglyceride

Triglyceride levels of serum were measured by DRI-CHEM SLIDE (470–03,701, Fujifilm Corporation, Tokyo, Japan) and DRI-CHEM auto tips (472–91,791, Fujifilm) using DRI CHEM 7000 V (Fujifilm).

### Measurement of serum electrolytes

Sodium, potassium, and chloride levels of serum were measured with an ion electrode method using a TBA-400FR Accute RX (Canon Medical Systems, Tochigi, Japan). The standard solution used was ISE CRS (J00700, Joko Co., Tokyo, Japan).

### Measurement of plasma CNP concentration

Plasma CNP levels were measured with an NPPC ELISA kit for mouse (OKEH03207, Aviva Systems Biology, San Diego, CA, USA) according to the manufacturer’s instructions.

### Urinalysis

Assay was performed using test papers (E-UR75, Eiken Chemical Co.,LTD, Tokyo, Japan). Urine was directly corrected by needle (29G SS-10M2913A, Terumo, Tokyo, Japan) under anesthesia. Each item was evaluated by dropping 10 µL of urine per item.

### Statistical analysis

Data are expressed as a mean ± standard deviation (SD). A survival curve and dot plots were generated using GraphPad Prism7 software (GraphPad Software, San Diego, CA, USA). Area of ICC, enteric neurons, blood vessel and ICC-IM in image field and gonadal adipose white tissue (GWAT) diameter were calculated by Fiji software^40^. The statistical significance of differences in mean values was assessed by Student’s *t*-test.

## Supplementary information

Supplementary information

## Data Availability

The data are available from the corresponding authors upon request.
