# Supplementary material for: Defective development and microcirculation of intestine in Npr2 mutant mice
Source: Sci Rep. 2020 Sep 8;10:14761. doi: 10.1038/s41598-020-71812-2 (PMC7479618; doi:10.1038/s41598-020-71812-2)
Supplement: Supplementary file 1 — Supplementary information [file 41598_2020_71812_MOESM1_ESM.pdf]

Title

Defective development and microcirculation of intestine in *Npr2* mutant mice

Authors

Chizuru Sogawa-Fujiwara[1*], Atsuki Hanagata[1], Yasuhiro Fujiwara[2*], Yukisato Ishida[3], Hirotaka Tomiyasu[4], Tetsuo Kunieda[5], Hirofumi Nakatomi[6], and Masatoshi Hori[1]

Affiliations

1, Veterinary Pharmacology, Graduate School of Agriculture and Life Sciences, The University of Tokyo, 1-1-1 Yayoi, Bunkyo-ku, Tokyo, Japan

2, Institute for Quantitative Biosciences, The University of Tokyo, 1-1-1 Yayoi, Bunkyo-ku, Tokyo, Japan

3, Graduate School of Human Health Sciences, Tokyo Metropolitan University, Minami-Osawa, Hachioji, Tokyo, Japan

4, Veterinary Internal Medicine, Graduate School of Agricultural and Life Sciences, The University of Tokyo, 1-1-1 Yayoi, Bunkyo-ku, Tokyo, Japan

5, Graduate School of Environmental and Life Sciences, Okayama University, Tsushima-naka, Kita-ku, Okayama, Japan

6, Department of Neurosurgery, Graduate School of Medicine, The University of Tokyo, 7-3-1 Hongo, Bunkyo-ku, Tokyo, Japan

[*] **Corresponding authors**:

chizuru.f.sogawa@gmail.com

y.fujiwara@iqb.u-tokyo.ac.jp

Defective development and microcirculation of intestine in *Npr2* mutant mice

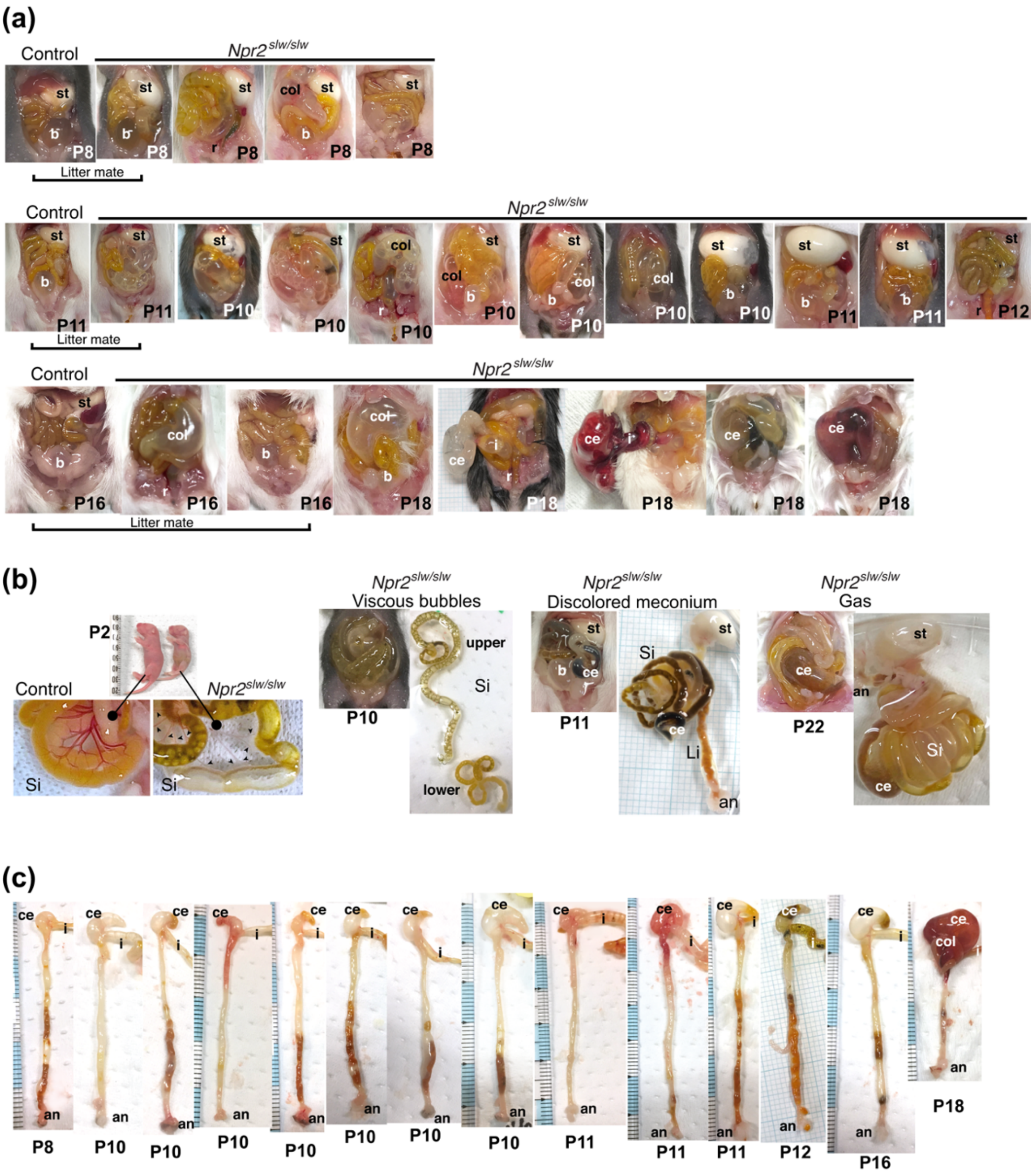

Supplementary Figure 1. GI phenotype in preweaning *Npr2*$^{slw/slw}$ mice.

(**a**) Autopsy images of preweaning control (leftmost) and *Npr2*$^{slw/slw}$ mice at various postnatal day. st: stomach; b: bladder; col: colon; ce: cecum; i: ileum; r: rectum. (**b**) Typical small intestine of *Npr2*$^{slw/slw}$ mice. Si: small intestine; Li: large intestine; st: stomach; b: bladder; ce: cecum; an: anus. (**c**) Large intestine of preweaning *Npr2*$^{slw/slw}$ mice. i: ileum; ce: cecum; an: anus.

Defective development and microcirculation of intestine in *Npr2* mutant mice

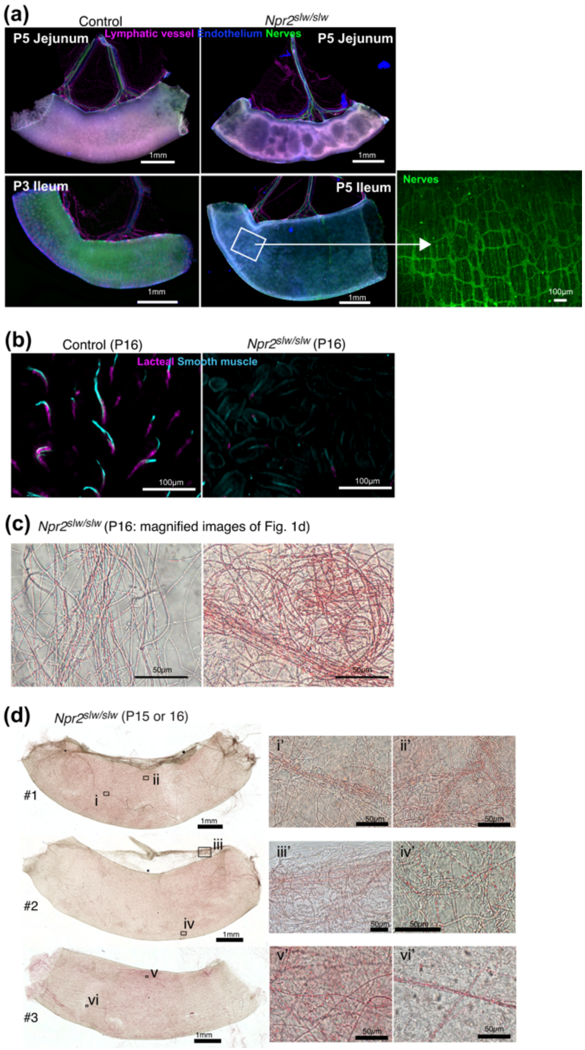

Supplementary Figure 2. Immunostaining and Oil Red-O staining

(**a**) Whole mount immunostaining of tube specimen. Low magnified images of immunostaining of jejunum and ileum from Control (left) and *Npr2*$^{slw/slw}$ (right) mice. PGP9.5 (green), LYVE1 (magenta), and PECAM (blue) were to identify ENS, lymphatic vessel and macrophage, and blood vessel, respectively. Bars are 1 mm. Magnified images of boxed region at border of stenosis and distention region showed PGP9.5 (green) to identify ENS. Bars are 100 μm. (**b**) Whole mount immunostaining of flat specimen of villus in jejunum at P16. LYVE1 (magenta) and aSMA (cyan) were to identify lacteal and smooth muscle of villus, respectively. Bars are 100 μm. (**c**) Whole mount Oil Red-O staining of tube specimen. Magnified image of small intestine in *Npr2*$^{slw/slw}$ mouse from Fig. 1d. Bars are 50 μm. (**d**) Whole mount Oil Red-O staining of tube specimen. *Npr2*$^{slw/slw}$ at P15 or 16. i' to vi' show magnified region of i to vi. Bars are 1 mm or 50 μm as indicated.

Defective development and microcirculation of intestine in *Npr2* mutant mice

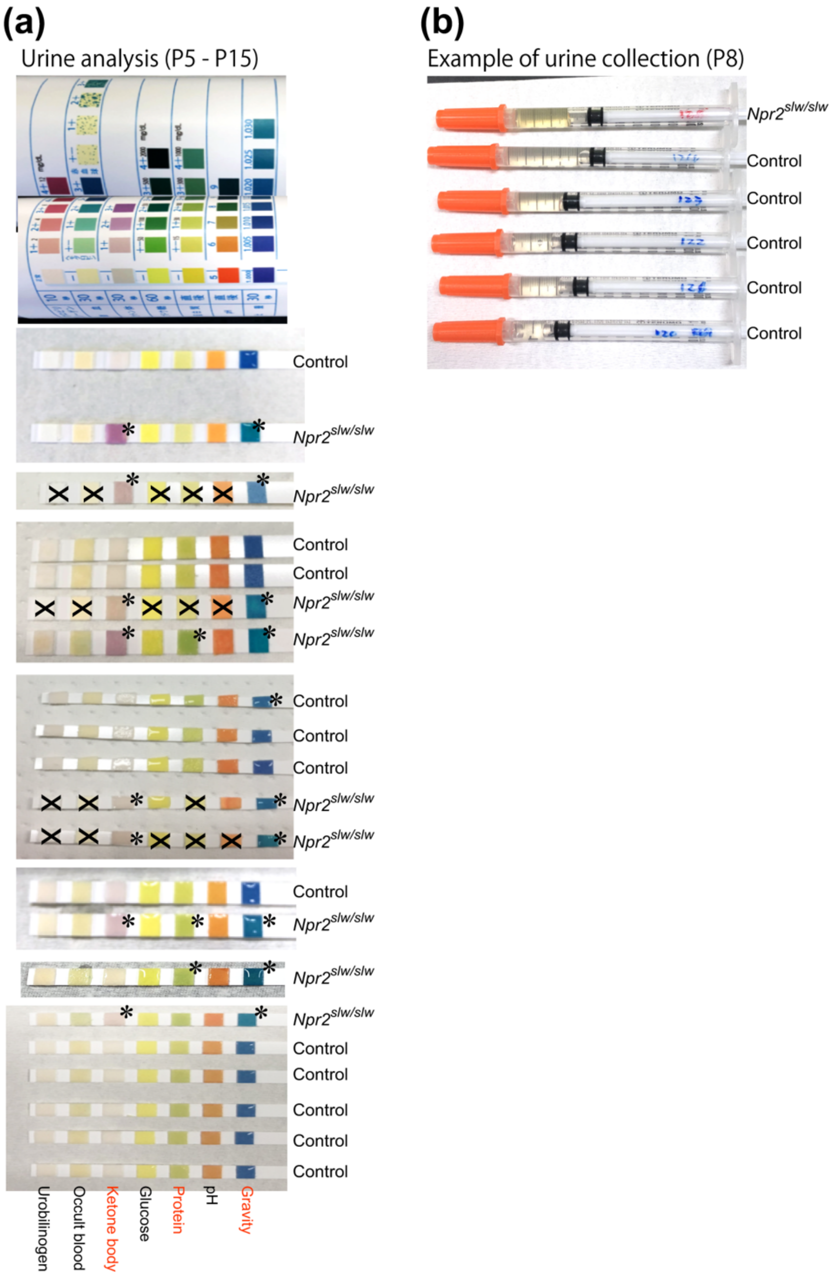

Supplementary Figure 3. Raw images of urinalysis

(**a**) Urinalysis used for Fig. 2A. Cross marks indicate non-dropped urine items. Asterisks indicate items with high index. (**b**) Examples of urine collection from P8 littermate.

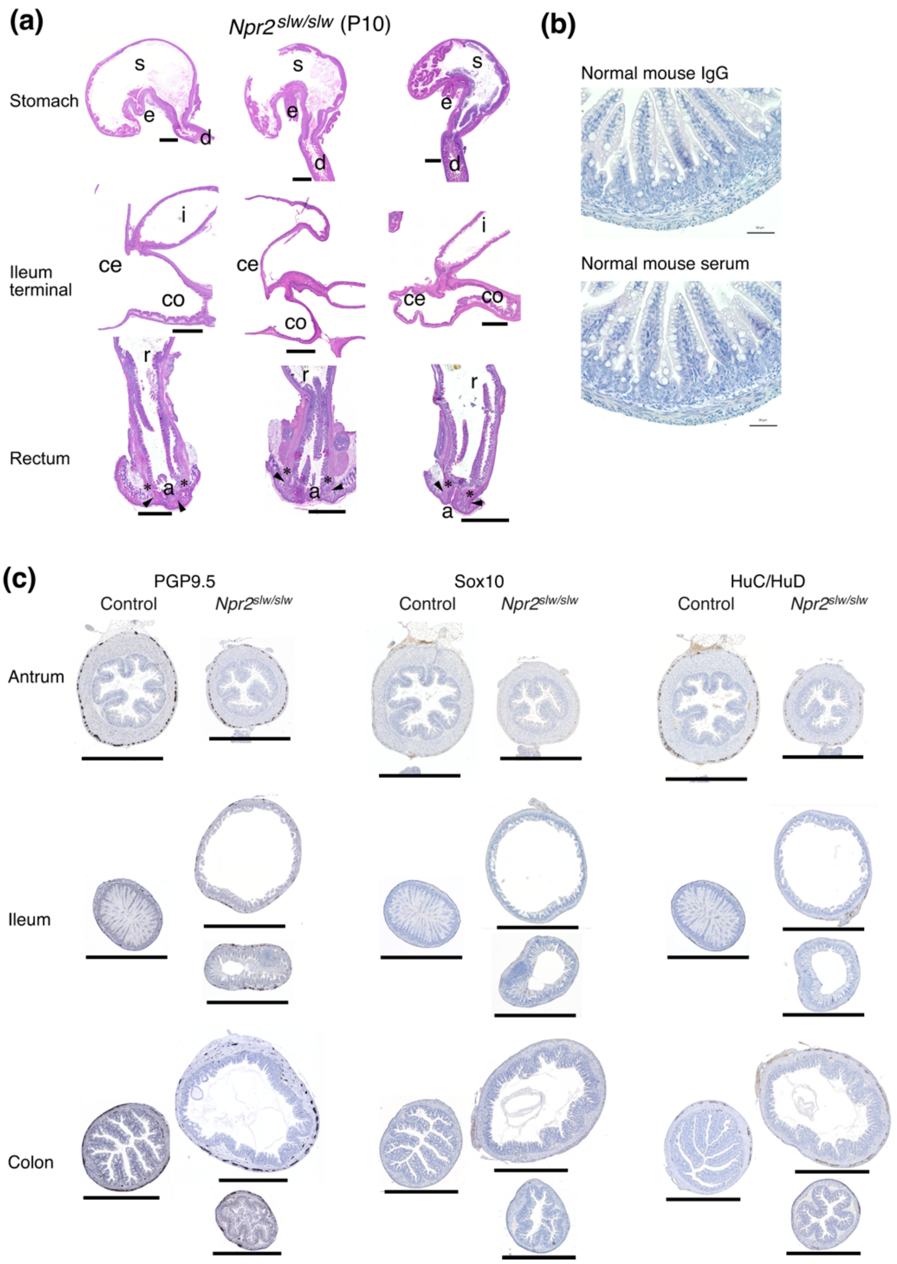

Supplementary Figure 4. Histological morphology in preweaning *Npr2*$^{slw/slw}$ mice.
(**a**) HE staining of vertical-sections of stomach to duodenum, terminal ileum area, and rectum to anus (S: stomach; e: esophagus; d: duodenum; i: ileum; ce: cecum; co: colon; r: rectum; a: anus) in *Npr2*$^{slw/slw}$ mice. Asterisks indicate internal anal sphincter and arrowheads indicate external anal sphincter in anus. Bars are 1 mm. (**b**) Negative control for immunohistochemistry of mouse antibodies. Bars are 50 µm. (**c**) Whole images of localization of neural protein (PGP9.5), enteric glia (Sox10), and ganglion cells (HuC/HuD) in cross-section of antrum, ileum, and proximal colon. Bars are 1 mm.

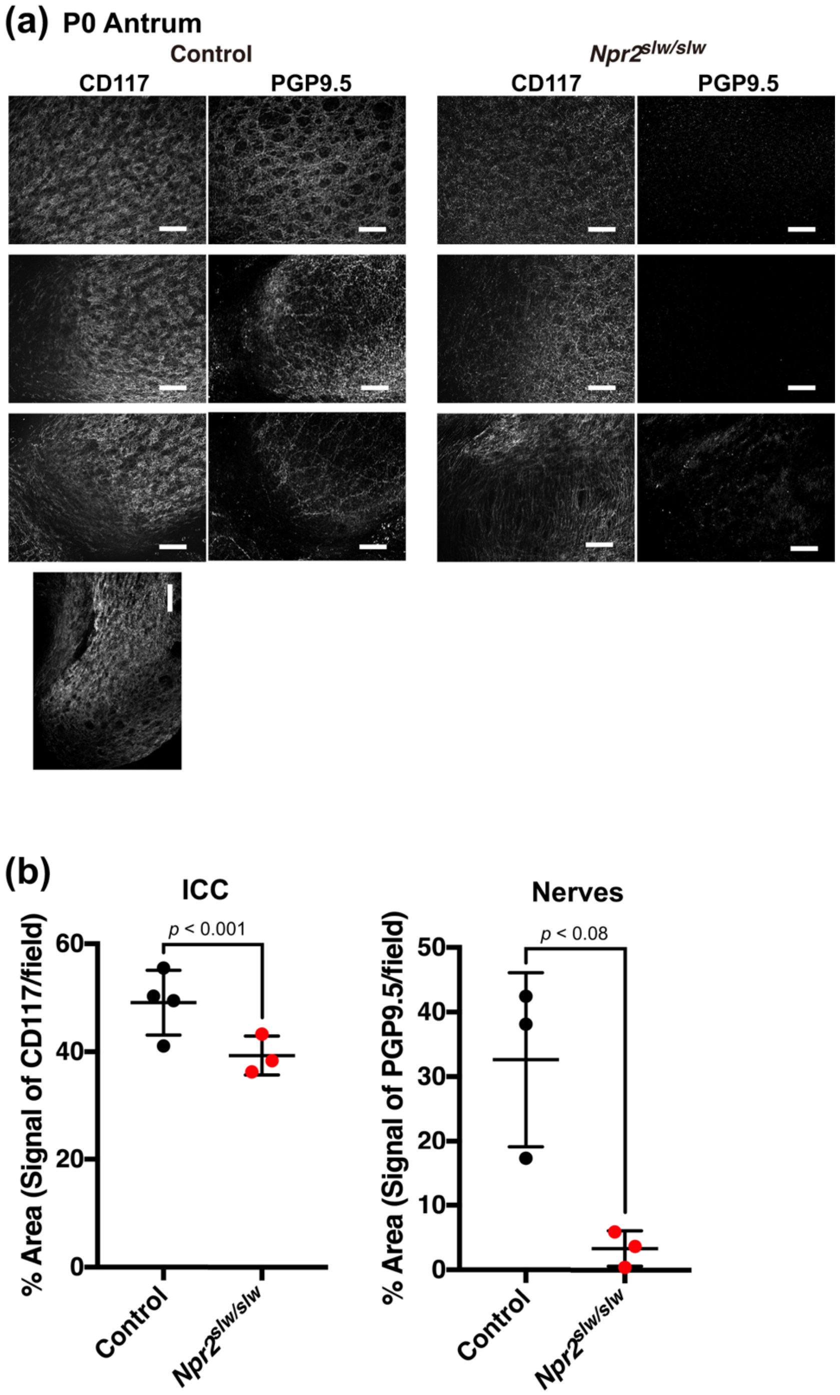

Supplementary Figure 5. Expression level of CD117 and PGP9.5 in antrum at newborn. (**a**) Whole mount immunostaining of tube specimens. Binarization image of CD117 and PGP9.5 signal in antrum. Control (left) and *Npr2*$^{slw/slw}$ (right) mice. Bars are 100 μm. (**b**) Percent of area of CD117 (left) and PGP9.5 (right) positive signal in image field (n=3 or 4 for control and *Npr2*$^{slw/slw}$ mice, respectively. Bars shown as mean ± SD).

Defective development and microcirculation of intestine in *Npr2* mutant mice

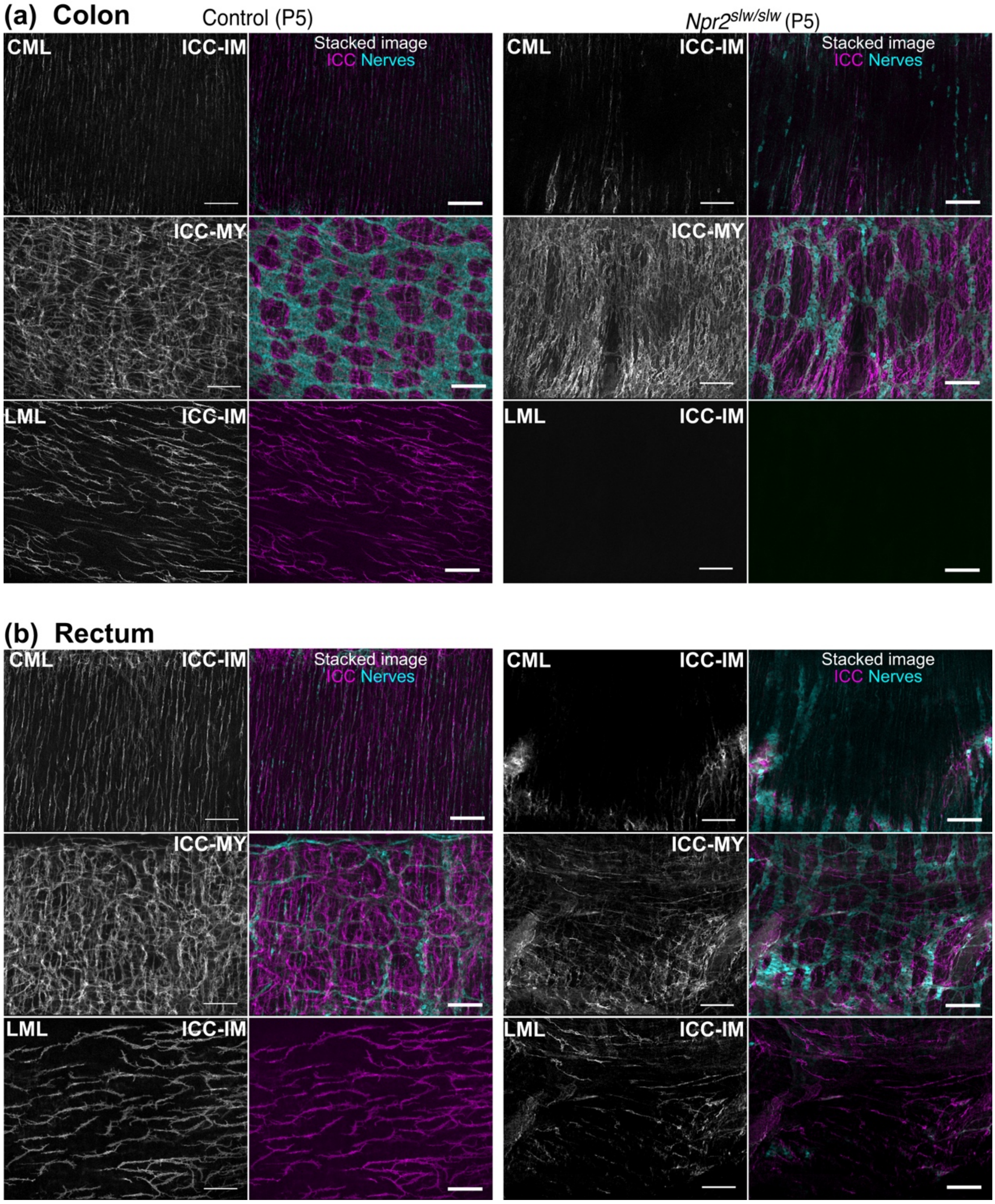

Supplementary Figure 6. Abnormality of ICC in large intestine of *Npr2*$^{slw/slw}$ mice.
Whole mount immunostaining of tube specimen. Immunostaining of PGP9.5 (green) and CD117 (magenta) were to identify ENS and ICC, respectively.
(**a**) proximal colon and (**b**) rectum of control and *Npr2*$^{slw/slw}$ mice at P5. CD117 in each layer were shown in grey as a single image and in magenta as a merged image with PGP9.5 (cyan). Bars are 100 µm. CML, circular muscle layer; ICC-MY, layer of Auerbach's plexus; LML, longitudinal muscle layer; ICC-IM, intramuscular ICC.

Defective development and microcirculation of intestine in *Npr2* mutant mice

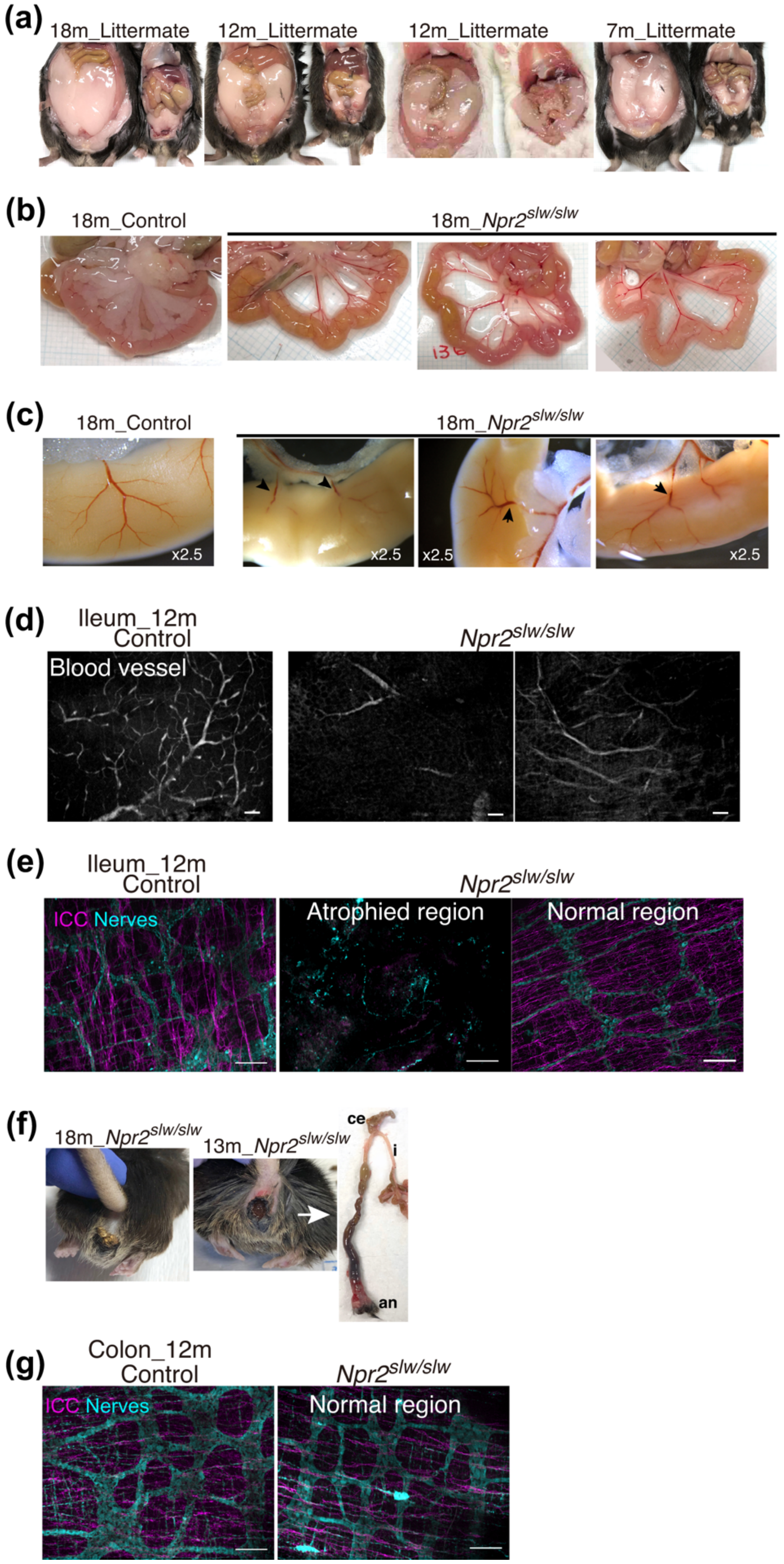

Supplementary Figure 7. Phenotype of adult mice. (**a**) Autopsy images of GWAT in control (left) and *Npr2*$^{slw/slw}$ (right) mice at different ages. (**b**) Mesenteric white adipose tissue in control (leftmost) and *Npr2*$^{slw/slw}$ mice. (**c**) Formalin fixed small intestine. Control (leftmost) and *Npr2*$^{slw/slw}$ mice. Arrow heads indicate swelling region of blood vessel. (**d**) Whole mount immunostaining of flat specimens of ileum. PECAM (gray) was to identify blood vessel of control and *Npr2*$^{slw/slw}$ mice, respectively. Bars are 100 µm. (**e**) Whole mount immunostaining of flat specimens of ileum. CD117 (magenta) and PGP9.5 (cyan) were to identify ICC and ENS of control and *Npr2*$^{slw/slw}$ mice, respectively. Bars are 100 µm. (**f**) Dyschezia in *Npr2*$^{slw/slw}$ mice and autopsy image of large intestine. i: ileum; ce: ceum; an: anus. (**g**) Whole mount immunostaining of flat specimens of colon. CD117 (magenta) and PGP9.5 (cyan) were to identify ICC and ENS of control and *Npr2*$^{slw/slw}$ mice, respectively. Bars are 100 µm.
